# A novel anatomic severity grading score for acute Type B aortic dissections and correlation to aortic reinterventions after thoracic endovascular aortic repair

**DOI:** 10.1186/s13019-017-0590-8

**Published:** 2017-05-23

**Authors:** Shirui Chen, Sebastian Larion, Sadaf S. Ahanchi, Chad P. Ammar, Colin T. Brandt, Jean M. Panneton

**Affiliations:** Division of Vascular Surgery, Department of Surgery, Eastern Virginia Medical School, Sentara Heart Hospital, 600 Gresham Drive, Suite 8620, Norfolk, VA 23507 USA

**Keywords:** Type B aortic dissection, Descending, Anatomic severity grading score, ASG, TEVAR, Endovascular repair

## Abstract

**Background:**

We introduce a novel preoperative anatomic severity grading system for acute type B aortic dissections and validate the system in a cohort of patients who underwent thoracic endovascular aortic repair.

**Methods:**

We identified a cohort of patients who received thoracic endovascular aortic repair (TEVAR) for acute type B aortic dissection from 2008 to 2014. We developed an anatomic severity grading score (ASG) to measure attributes of aortic anatomy that we hypothesized may affect difficulty or durability of repair. Measurements were made using computed tomography angiography images and based on hypothesized severity, giving a potential score range of 0-38.

**Results:**

We analyzed the computed tomography angiography images on a cohort of 30 patients with acute type B aortic dissection who underwent TEVAR. We created an area under the receiver operating characteristic curve (AUROC) using anatomic severity grading to predict aortic-related reinterventions. The AUROC was 0.72 (95% CI 0.39 to 1.1). Guided by the AUROC, we divided patients into two groups: a low-score group with anatomic severity grading scores <23 (*n* = 22), and a high-score group with scores ≥23 (*n* = 8). With this cutoff, anatomic severity grading exhibited 80% sensitivity and 84% specificity in predicting aortic-related reinterventions, with reinterventions in 50% of high-score patients and 4.5% of low-score patients (*P* = 0.011). The high score group also had significantly greater blood loss (200 vs 100 mL, *P* = 0.038), fluoroscopy time (36.0 vs 16.6 min; *P* = 0.022), and a trend for increased procedure time (164 vs 95 min; *P* = 0.083) than the low-risk group. Kaplan-Meier analysis revealed that the high-score group had a significantly decreased freedom from aortic-related reinterventions than the low-score group (38% vs 100% at 12-month followup; log rank *P* = 0.001).

**Conclusions:**

A preoperative anatomic severity grading score for acute type B aortic dissections consists of analysis of the proximal landing zone, curvature and tortuosity of the aorta, dissection anatomy, aortic branch vessel anatomy, and supraceliac aorta anatomy. Anatomic severity grading scores ≤23 are an excellent predictor of aortic-related reinterventions.

## Background

Aortic aneurysms and dissections are both commonly treated with endovascular repair. Aortic anatomic factors are known to affect procedural difficulty and patient selection for endovascular repair. Accordingly, the Society for Vascular Surgery (SVS) anatomic severity grading (ASG) system was developed to objectively assess the severity of abdominal aortic aneurysms (AAA) based on morphologic variables related to the aortic neck, iliac artery, pelvic perfusion and aortic aneurysm itself [1]. ASG score was found to correlate with a number of intraoperative and short term outcomes in patients who underwent endovascular aneurysm/aortic repair (EVAR) and fenestrated EVAR (FEVAR) for infrarenal and juxtarenal AAA [2, 3]. However, to our knowledge, there is no ASG system to help predict outcomes for endovascular treatment of aortic dissections.

In a review of 18 studies of predictors of aortic growth in uncomplicated type B aortic dissections (TBAD), aortic diameter ≥ 40 mm, proximal descending thoracic aorta false lumen diameter ≥ 22 mm, degree of fusiform dilation of the proximal descending aorta, elliptic shape of the true lumen, patency of the false lumen, partial thrombosis of the false lumen, saccular degeneration of the false lumen, presence of one entry tear, proximal entry tear ≥ 10 mm, location of the false lumen at the inner curvature of the aorta, and presence of ulcer-like projections were correlated with increasing aortic growth [4]. Using a review of the literature, in combination with expert opinion, we sought to develop an ASG scoring system for aortic dissections that is analogous to the SVS ASG score for AAAs. Therefore, the aim of this study was to create a novel ASG scoring system for patients with acute TBAD, and subsequently validate the system by analyzing its capability to identify patients at-risk for post-operative reintervention following TEVAR.

## Methods

We performed a retrospective review of patients undergoing TEVAR for acute TBAD at our institution from 2008 to 2014 using Current Procedural Terminology codes 33880 and 33881. Patients were included in the study if they underwent TEVAR within two weeks of symptoms onset. In addition, the most proximal extent of the false lumen had to be located in the area between and including aortic zone 2 (between the left common carotid artery [LCCA] and left subclavian artery [LSA]) and the distal-most boundary of the thoracic aorta (delineated by the aortic hiatus of the diaphragm). This included patients with thoracoabdominal, or DeBakey type IIIb, dissections. Additionally, the entry tear must have been located in zone 3 or zone 4 of the thoracic aorta (distal to the LSA). Patients were excluded if the dissection was chronic (>2 weeks), due to traumatic etiology, or associated with rupture. Patients were also excluded if aneurysmal dilation was greater than 6 cm in diameter (measured from outer wall-to-outer wall), or they had previously undergone thoracic aorta repair. Anatomic factors thought to potentially affect the difficulty and outcome of TEVAR for acute TBAD were assessed using 3D reconstructions of preoperative CT angiography data from patient medical records on a TeraRecon digital workstation (TeraRecon iNtuition Workstation; TeraRecon, Foster City, CA). These factors were selected based on review of the literature as well as expert opinion (Table 1). Factors were divided into five subgroups, each representing a general attribute of aortic anatomy thought to affect TEVAR difficulty and outcomes: proximal landing zone, curvature and tortuosity of the aorta, dissection anatomy, aortic branch vessel anatomy, and supraceliac aorta anatomy.

**Table 1:** Anatomic severity grading score subcomponent descriptions

	Attribute	Description
Proximal Landing Zone	Whole lumen cross-sectional area contour change (CC1)	The difference between the largest measured whole lumen cross-sectional area and the smallest, out of five measurements taken at standardized locations near the proximal landing zone.
	Maximum whole lumen cross-sectional area (mCSA1)	The largest whole lumen cross-sectional area out of five measurements taken at standardized locations near the proximal landing zone.
	Left common carotid to left subclavian distance (L1)	The centerline distance between the left common carotid and the left subclavian arteries
	Left common carotid to entry tear distance (L2)	The centerline distance between the left common carotid and the most proximal entry tear.^b^
	Calcification and thrombus index (CT)	The volume of thrombus plus calcification divided by the volume of unthrombosed aorta lumen from the LCCA to a level 20 mm distal to the LSA. Thrombus = 51-150 HU; Calcium = > 150 HU
Curvature and Tortuosity	Thoracic aorta tortuosity index (TT)	Ratio of centerline distance between left common carotid and superior mesenteric arteries over the straight-line distance between the same two locations
	Intraaortic distance (ID)	Half the minimum distance between the inner curvature of the ascending aorta to the inner curvature of the descending aorta at the most superior level at which the bifurcation of the pulmonary artery can be observed.
	Arch type	Vertical distance from the (middle of the) origin of the innominate artery to the top of the arch (Inn-Top). Type I: < 1 diameter of the LCCA. Type II: between 1 and 2 LCCA diameters. Type III: > 2 LCCA diameters.
Dissection	False lumen cross-sectional area index (FCI)	The ratio between the cross-sectional area of the false lumen and that of the sum of both the false and true lumens, measured 20 mm distal to the LSA.
	False lumen length (FLL)	Centerline measurement from proximal extent of false lumen to distal extent of false lumen, including thrombosed portions.
	Tear length	Length of largest entry tear measured orthogonal to the centerline.^b^
	Number of observable tears	
	Tear circumferential location	Location of the tear^b^
Branch Vessels^a^	Location (BVL)	Number of branch vessels whose ostia are located partially or entirely in the false lumen
	Patency (BVP)	BVP is based on an occlusion index (OI), where a branch vessel is assigned an index of 0 if the ostium is patent, 1 if partially occluded and 2 if completely occluded. BVP for each patient is then categorized based on the summed occlusion indices of all branch vessels.
Supraceliac Aorta Anatomy	Whole lumen cross-sectional area contour change (CC2)	The difference between the largest measured whole lumen cross-sectional area and the smallest, out of eight measurements taken at standardized locations on the supraceliac aorta.
	Maximum whole lumen cross-sectional area (mCSA2)	The largest whole lumen cross-sectional area out of eight measurements taken at standardized locations on the supraceliac aorta.

^a^Branch vessels examined: left subclavian artery, celiac artery, superior mesenteric artery, left renal artery, right renal artery, inferior mesenteric artery, left iliac artery, right iliac artery

^b^If an entry tear was not observed the patient received a score of 0 for this attribute

**Fig. 1:**
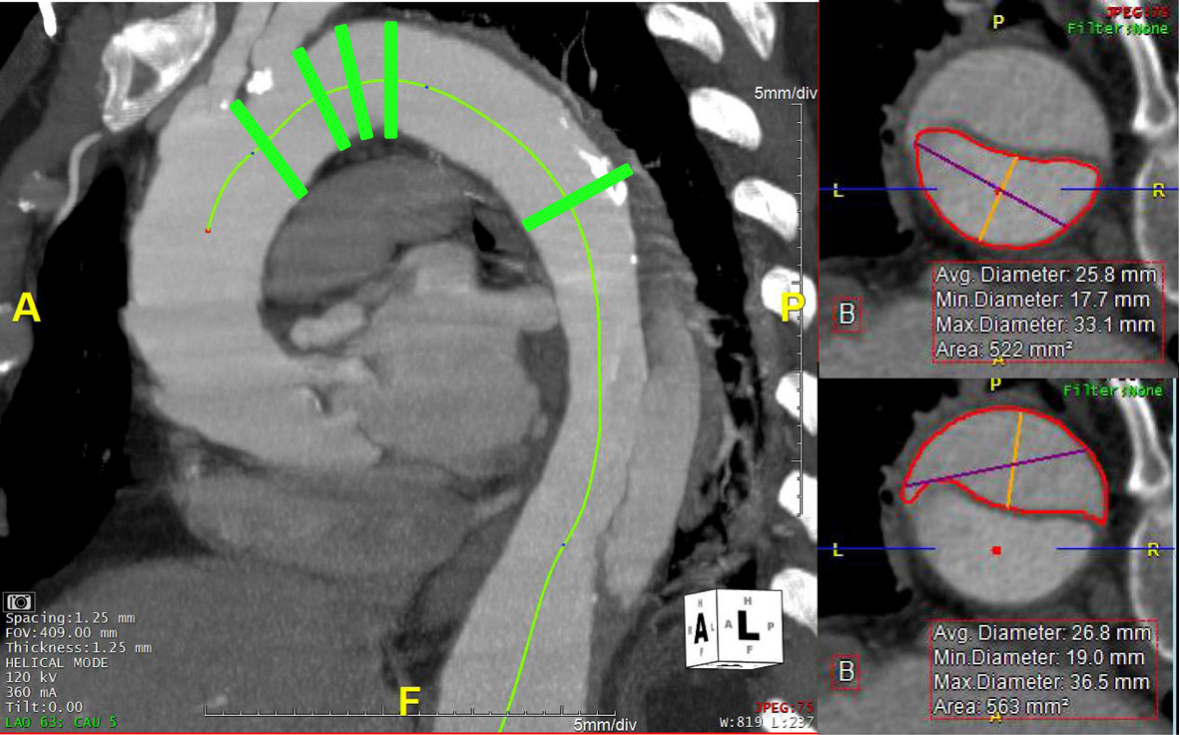
Proximal landing zone. Green bars (*left*) denote locations at which cross-sectional area measurements were made of the true and false lumens. At right, the process of measuring cross-sectional area of the true lumen (*top*) and false lumen (*bottom*) are shown in representative sections of the aorta

The proximal landing zone subgroup assesses several factors, including cross-sectional area, length, and calcification and/or thrombus burden. Cross-sectional areas of the true and false lumens were measured at five locations within or near the proximal landing zone: immediately distal to the LCCA, immediately distal to the LSA, 10 mm and 20 mm distal to the edge of the LSA ostium, and at the most proximal extent of the false lumen (Fig. 1). The volume of calcification and thrombus was measured based on Hounsfield units.

**Fig. 2:**
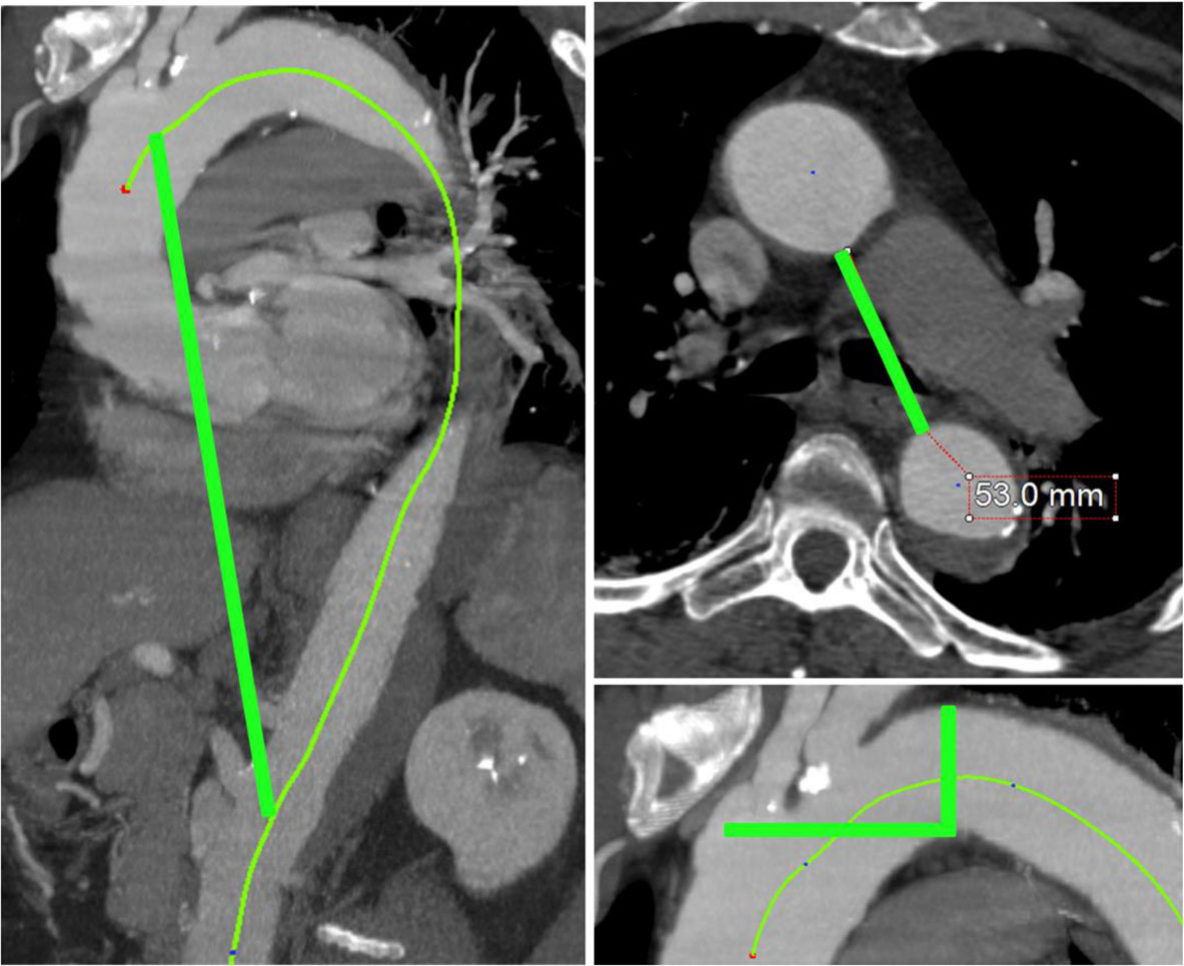
Curvature and tortuosity. At left, the thoracic aorta tortuosity index is determined by taking the ratio of the center lumen line distance from the ostium of left common carotid artery to the ostium of the superior mesenteric artery, and the straight line distance between the same two points. At top right, the intraaortic distance is calculated as half the minimum distance between the inner curvature of the ascending aorta to the inner curvature of the descending aorta at the most superior level at which the bifurcation of the pulmonary artery can be observed. At bottom right, arch type is determined by the vertical distance from the (middle of the) origin of the innominate artery to the top of the arch. Type I: < 1 diameter of the LCCA. Type II: between 1 and 2 LCCA diameters. Type III: > 2 LCCA diameters

The curvature and tortuosity subgroup captures aortic arches of narrow radii as well as aortas that present with more tortuous anatomy (Fig. 2).

The dissection subgroup measures anatomic factors associated with the dissection itself, specifically the entry tear and the length of the dissection. It also includes measurements of the cross sectional area of the false lumen in relation to that of the aortic lumen as a whole.

The branch vessel subgroup captures the presence of major aortic branch vessels within the dissection territory.

**Fig. 3:**
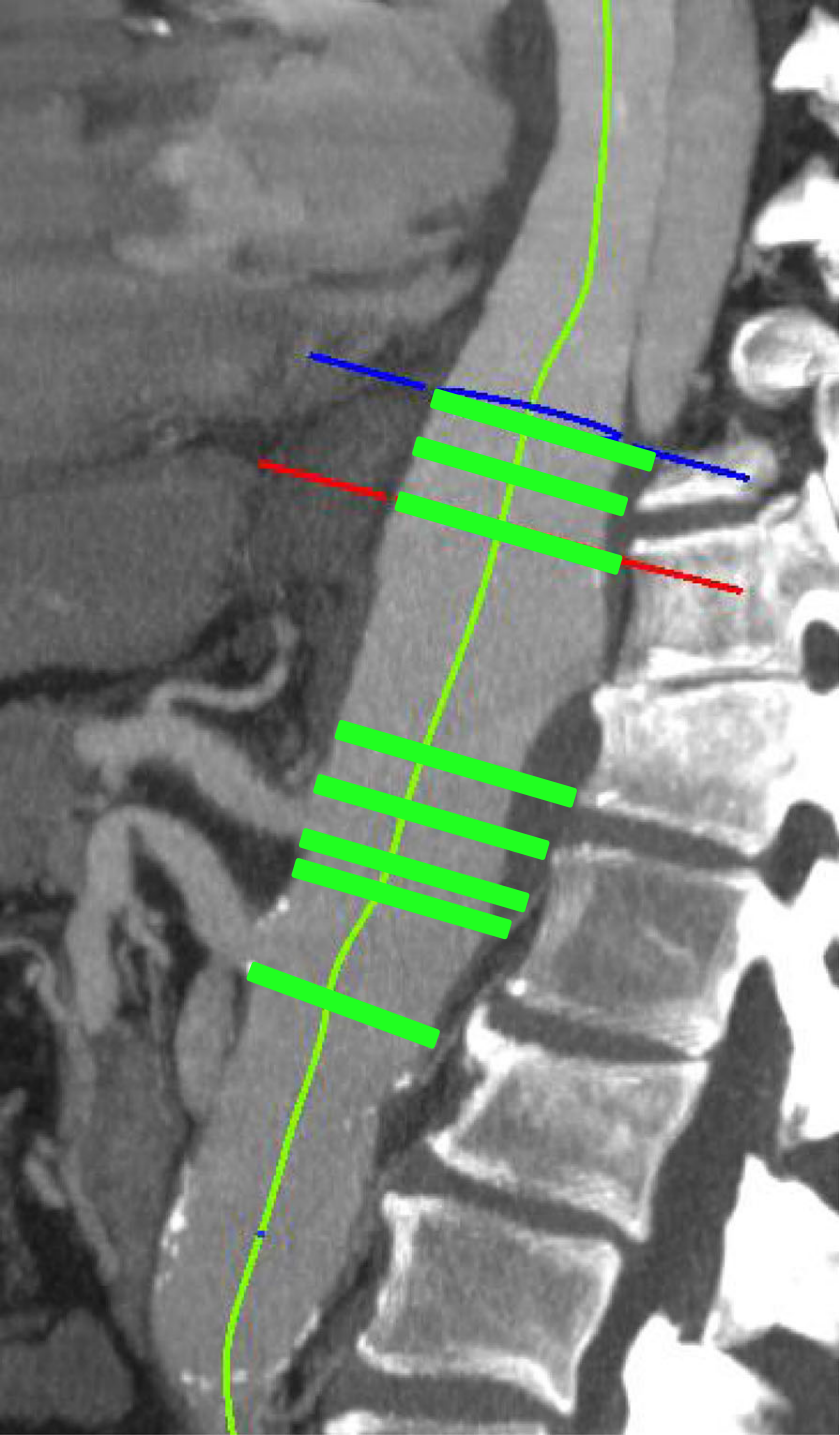
Supraceliac aorta. Green bars denote locations at which cross-sectional area measurements were made of the true and false lumens

Finally, the supraceliac aorta subgroup captures anatomy in the supraceliac aorta, such as aortic cross-sectional area. Cross-sectional area of true and false lumens were measured at eight standardized locations for the supraceliac subscore: 20 mm and 10 mm proximal to the aortic hiatus, at the level of the aortic hiatus, 20 mm and 10 mm proximal to the distal edge of the celiac artery ostium, at the distal edge of the celiac artery ostium, at the distal edge of the superior mesenteric artery (SMA) ostium, and at the most distal extent of the false lumen (Fig. 3).

After imaging data was collected, individual factors were assigned points with cutoff values that took into consideration the distribution of morphological measurements (Table 2). Measurements were categorized using scores of 0 to 3 based on increasing hypothesized severity. Individual measurements are then summed for each patient, giving a potential range of 0-38.

**Table 2:** Anatomic severity grading scoring system

	Attribute	0	1	2	3
Proximal Landing Zone	Whole lumen cross-sectional area contour change (CC1)	<200 mm^2^	200 - 450 mm^2^	450 - 700 mm^2^	>700 mm^2^
	Maximum whole lumen cross-sectional area (mCSA1)	<1000 mm^2^	1000 - 1100 mm^2^	1100 - 1400 mm^2^	>1400 mm^2^
	Left common carotid to left subclavian distance (L1)	>18 mm	18 - 12 mm	<12 mm	
	Left common carotid to entry tear distance (L2)	>100 mm	100 - 25 mm	<25 mm	
	Calcification and thrombus index (CT)	<0.3	0.3 - 0.5	>0.5	
Curvature and Tortuosity	Thoracic aorta tortuosity index (TT)	<1.25	1.25 - 1.35	>1.35	
	Intraaortic distance (ID)	>28 mm	28 - 23 mm	<23 mm	
	Arch type	Type I	Type II	Type III	
Dissection	False lumen cross-sectional area index (FCI)	<0.2	0.2 - 0.5	0.5 - 0.65	>0.65
	False lumen length (FLL)	<300 mm		>300 mm	
	Tear length	<5 mm	5 - 17 mm	>17 mm	
	Number of observable tears	0	>0		
	Tear circumferential location	Not at outer curvature of arch	At outer curvature of arch		
Branch Vessels	Location (BVL)	0	1 - 3	4	>4
	Patency (BVP)	0	1 - 2	>2	
Supraceliac Aorta Anatomy	Whole lumen cross-sectional area contour change (CC2)	<300 mm^2^	300 - 400 mm^2^	400 - 600 mm^2^	>600 mm^2^
	Maximum whole lumen cross-sectional area (mCSA2)	<650 mm^2^	650 - 850 mm^2^	850 - 925 mm^2^	>925 mm^2^

We also sought to correlate the ASG score with the volumes of the false and true lumen. The volumes of the false and true lumens were measured from the LSA to the celiac artery. The cross-sectional areas of the false and true lumens were measured at 2 cm intervals, and volume was calculated as the sum of the volumes of a series of irregular cylinders [5]. A false lumen volume index (FLVI = false lumen volume divided by the sum of the true and false lumen volumes) was then calculated.

An area under the receiver operating characteristic curve was created to determine patients at risk for aortic-related reinterventions. Patients were divided into a low-score (ASG < 23) and high-score (ASG ≥ 23) group based upon their total ASG score. Chi-square and Fisher’s exact test were used to determine differences in proportions of categorical variables, as appropriate. Analysis of variance was used to determine differences in means of continuous variables.

Aortic reinterventions and thoracic aorta false lumen volume index were additionally assessed by multivariate analysis. Multiple logistic regression was used to identify anatomic factors that were independently associated with aortic reinterventions, where factors significant (*P* < 0.05) on univariate analysis were included in the logistic model. Multiple linear regression using the forward stepwise method was used to identify parameters that could predict thoracic aorta false lumen volume index as the dependent variable. Anatomic scoring parameters were inputted as covariates with the minimum F-to-enter set at 4.0.

All data analysis was performed using IBM SPSS version 22.0 (IBM Corp: Armonk, NY) and SigmaPlot v12.0 (San Jose, CA).

## Results

A total of 30 patients satisfied the inclusion criteria and were included in the final analysis. Patient demographics are listed in Table 3, showing that most patients were male (70%) with a high prevalence of hypertension (90%) and smoking history (63%). Patients in the high-score group were significantly younger than patients in the low-score group (54 vs 66 years; *P* = 0.027). There were no significant differences in medical comorbidities between patient groups (*P* > 0.05; Table 3). The mean interval between onset of dissection symptoms (i.e. chest or back pain, dyspnea, diaphoresis) and TEVAR was 4.2 days.

**Table 3:** Patient medical comorbidities between low and high score groups

	Low score (*n* = 22)	High score (*n* = 8)	*P*-value
Age (years)^a^	65.5 ± 11.8	54.1 ± 12.0	0.027
Male	68%	75%	1.000
Caucasian	45%	63%	0.682
Coronary artery disease	23%	0%	0.287
Cerebrovascular accident	9%	13%	1.000
Congestive heart failure	9%	13%	1.000
Arrhythmia	9%	13%	1.000
Hypertension	86%	100%	0.545
Dyslipidemia	36%	38%	1.000
Diabetes mellitus type 2	18%	0%	0.550
Chronic obstructive pulmonary disease	18%	0%	0.550
Chronic kidney disease	9%	25%	0.284
Dialysis	5%	13%	0.469
Body mass index (kg/m^2^)^a^	28.5 ± 5.4	33.7 ± 8.3	0.076
History of smoking	64%	63%	1.000

^a^Data shown is standard deviation

Anatomic variables relating to aortic morphology for the entire study cohort are listed in Table 4. At 20 mm distal to the LSA, mean true lumen diameter was 28.4 mm (SD: 4.4, range: 19.4-40.3 mm), mean false lumen diameter was 33.1 mm (SD: 14; range: 8.0-54.2 mm), and mean thoracic aorta FLVI was 0.59 (range: 0.19-0.94). The most commonly implanted endografts were Talent© (43%), Valiant© (33%; Medtronic, Santa Rosa, CA), Cook TX2© (17%; Cook Medical, Bloomington, IN), and Gore C-TAG© (7%; WL Gore & Associates, Flagstaff, AZ). Technical success was achieved in 100% of TEVARs, with a mean of 2.0 pieces implanted per patient (range: 1-4 pieces). Mean duration of procedure was 124 min (range: 58-248 min); mean contrast volume was 162 mL, and mean estimated blood loss was 217 mL.

**Table 4:** Anatomic variables of study cohort

Variable group	Variable^a^	Low ASG mean ± SD	High ASG mean ± SD	All subjects mean ± SD	*P*-value^c^
Proximal Landing Zone	Whole lumen cross-sectional area contour change (CC1)	285 ± 139	824 ± 405	429 ± 335	**0.007**
	Maximum whole lumen cross-sectional area (mCSA1)	935 ± 189	1546 ± 300	1097 ± 351	**0.0004**
	Left common carotid to left subclavian distance (L1)	16.2 ± 5.5	12.9 ± 3.9	15.3 ± 5.3	0.17
	Left common carotid to entry tear distance (L2)	79.3 ± 68.3	27.6 ± 13.7	65.9 ± 63.1	0.21
	Thrombus^b^	6.3 ± 2.1	10.1 ± 6.7	7.3 ± 4.0	0.15
	Calcification^b^	2.2 ± 0.9	2.0 ± 1.1	2.2 ± 1.0	0.56
	Total unthrombosed lumen^b^	23.7 ± 8.3	28.3 ± 14.4	24.9 ± 10.2	0.42
Curvature and Tortuosity	Center lumen line from left common carotid artery to superior mensenteric artery	301 ± 27.8	304 ± 25.1	302 ± 26.6	0.84
	Straight Line from left common carotid artery to superior mensenteric artery	225 ± 18.1	244 ± 19.4	230 ± 19.9	**0.040**
	Intraaortic Distance (ID)	24.7 ± 5.3	25.4 ± 4.5	24.9 ± 5.0	0.72
	Left common carotid diameter	15.5 ± 27.1	12.0 ± 6.3	14.6 ± 23.3	0.58
	Vertical distance from the origin of the innominate artery to the top of the arch (Inn-Top)	24.6 ± 11.4	22.6 ± 11.5	24.0 ± 11.2	0.68
Dissection	True lumen diameter 20 mm distal to LSA	28.7 ± 5.1	27.6 ± 2.0	28.4 ± 4.4	0.42
	False lumen diameter 20 mm distal to LSA	28.4 ± 13.3	45.9 ± 4.7	33.1 ± 14.0	**0.00001**
	False lumen cross sectional area 20 mm distal to LSA	382 ± 225	1138 ± 392	583 ± 435	**0.0007**
	True lumen cross sectional area 20 mm distal to LSA	477 ± 257	344 ± 149	442 ± 239	0.093
	False lumen length (FLL)	306 ± 97	384 ± 90	327 ± 100	0.058
	Tear length	12.0 ± 6.8	18.8 ± 7.3	13.8 ± 7.4	0.058
	Number of observable tears	1.14 ± 0.71	2.00 ± 1.7	1.37 ± 1.1	0.20
	Tear circumferential location subscore	0.68 ± 0.48	0.75 ± 0.46	0.7 ± 0.47	0.73
Branch Vessels	Branch vessel location subscore (BVL)	0.82 ± 1.1	1.6 ± 1.3	1.03 ± 1.2	0.14
	Branch vessel patency subscore (BVP)	0.50 ± 0.67	0.63 ± 0.92	0.53 ± 0.73	0.73
Supraceliac Aorta Anatomy	Whole lumen cross-sectional area contour change (CC2)	402 ± 157	527 ± 277	436 ± 199	0.26
	Maximum whole lumen cross-sectional area (mCSA2)	777 ± 201	909 ± 370	812 ± 257	0.36

^a^All lengths are listed in millimeters, and all areas are listed in millimeters squared. Bolded *P*-values indicate* P* < 0.05

^b^Centimeters cubed

^c^Between the low and high-score patient groups

The mean hospital length of stay for the study cohort was 16.7 days (range: 1-40 days). Three patients (10%) died perioperatively, defined as within 30 days of the procedure. Three patients (10%) had radiological evidence and clinical features of cerebrovascular accident postoperatively. No patients experienced paraplegia. However, two patients experienced lower extremity paresis that did not fully resolve by discharge.

The average length of patient follow-up was 12.0 months (range: 1 day to 67 months). The all-cause mortality rate was 17% (*n* = 5). The aortic-related mortality rate was 3% (*n* = 1) and involved a patient who died 15 months after the index TEVAR due to a rupture of an anastomotic aneurysm from an open AAA repair performed years prior to the TEVAR. The stroke-related mortality was 7% (*n* = 2). Over the length of the study period, 17% (*n* = 5) of patients required aortic-related reinterventions: one patient developed a type 1a endoleak requiring proximal extension, one patient developed a symptomatic descending penetrating atherosclerotic ulcer distal to the existing graft requiring distal extension, one endograft experienced a partial collapse requiring additional proximal stent placement, one patient developed arm ischemia requiring LCCA to LSA bypass with brachial embolectomy, and one patient underwent a repeat TEVAR for a distal extension of the original dissection. The mean interval between the index TEVAR and the aortic-related reintervention was 126 days (range 8-544, median 17).

**Fig. 4:**
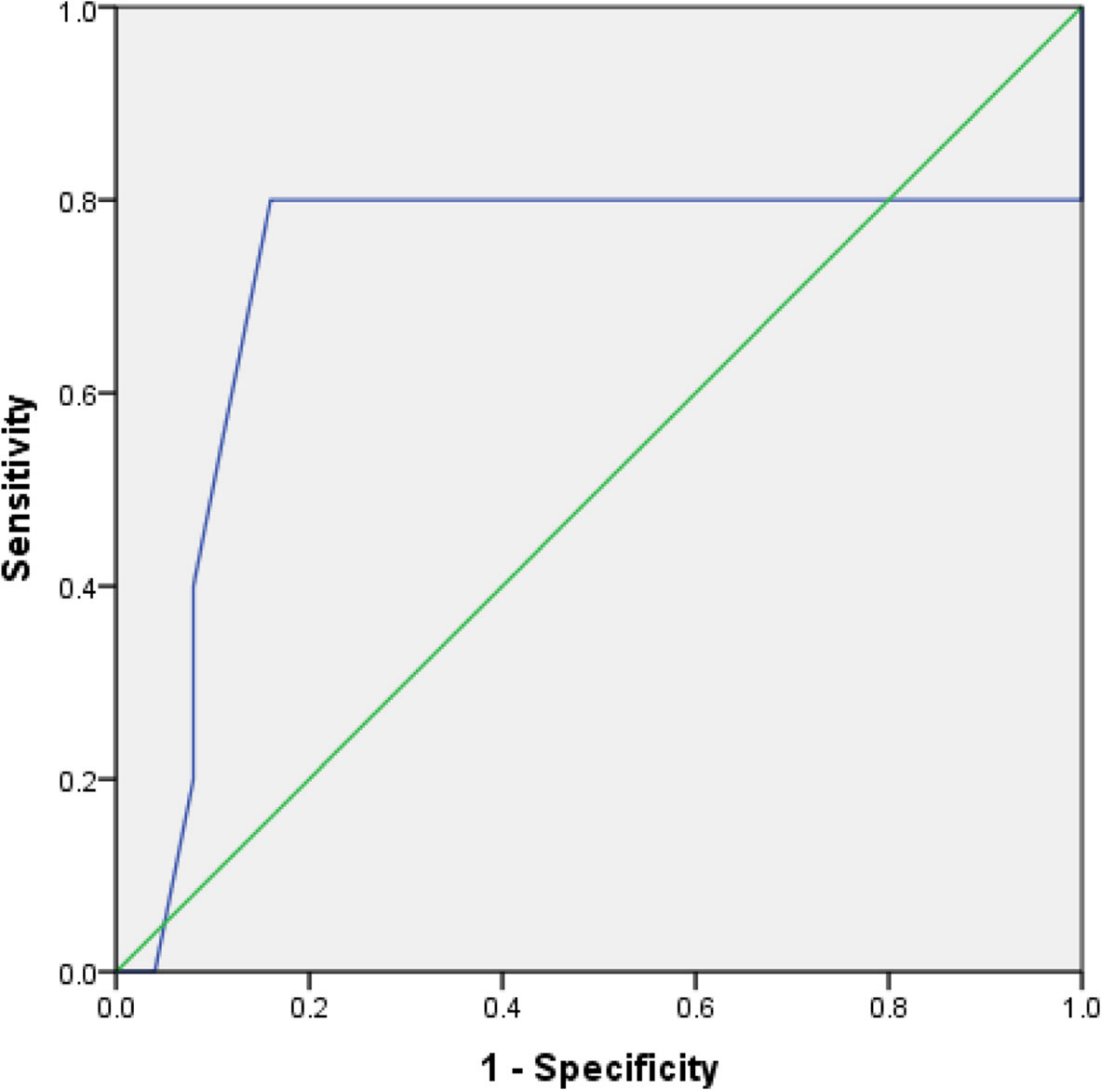
Receiver-operating characteristic curve using ASG score to predict aortic-related reinterventions (area = 0.72)

An area under the receiver operating characteristic (AUROC) curve was created to assess the predictive capability of using an ASG score to predict patients at risk for aortic-related reinterventions (Fig. 4). The receiver operating characteristic showed fair discriminatory power in identifying patients at high risk for aortic-related reinterventions with an area of 0.72 (95% CI: 0.39-1.1). Guided by the AUROC, patients were divided into a low and high-score patient group based upon an ASG cutoff score of 23 (sensitivity: 80%; specificity: 84%; positive likelihood ratio: 5.00; negative likelihood ratio: 0.238). This resulted in 22 patients in the low-score group and 8 patients in the high-score group. Complete receiver operating characteristic curve analysis can be found in Table 5.

**Table 5:** Detailed receiver-operating characteristic curve analysis

ASG greater than or equal to:	Sensitivity	Specificity	Positive likelihood ratio	Negative likelihood ratio
9	100%	0%		
10.5	80%	0%	0.800	
11.5	80%	4%	0.833	5.000
12.5	80%	12%	0.909	1.667
13.5	80%	16%	0.952	1.250
14.5	80%	32%	1.177	0.625
16	80%	36%	1.250	0.556
17.5	80%	40%	1.333	0.500
18.5	80%	52%	1.667	0.385
19.5	80%	60%	2.000	0.333
20.5	80%	68%	2.500	0.294
21.5	80%	76%	3.333	0.263
22.5	80%	84%	5.000	0.238
23.5	40%	92%	5.000	0.652
24.5	20%	92%	2.500	0.870
27	0%	96%	0	1.042
30	0%	100%		

*ASG* anatomic severity grading

**Fig. 5:**
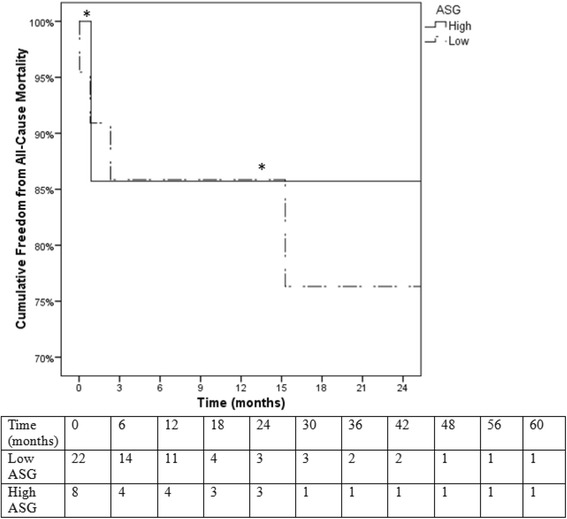
Kaplan-Meier survival analysis for all-cause mortality. There were no significant differences in all-cause mortality between the high-ASG and low-ASG groups (log rank *P* = .80). *Standard error of the mean > 10%

**Fig. 6:**
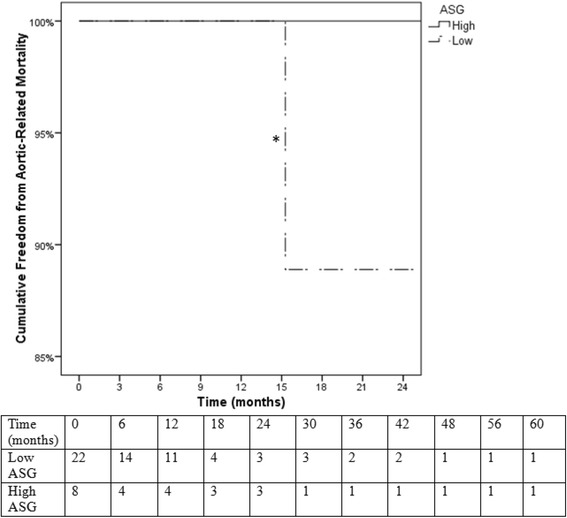
Kaplan-Meier survival analysis of aortic-related mortality. There were no significant differences in aortic-related mortality between the high-ASG and low-ASG groups (log rank *P* = .56). *Standard error of the mean > 10%

**Fig. 7:**
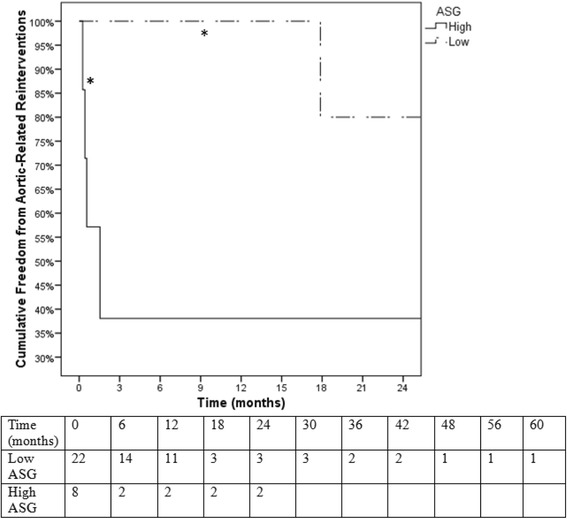
Kaplan-Meier survival analysis of aortic-related reinterventions. Patients in the high-ASG score group exhibited significantly greater aortic-related reinterventions than patients in the low-score group (log rank *P* = .001). *Standard error of the mean > 10%

Surgical data and patient outcomes were compared between low and high-score ASG groups. The median number of implanted pieces was no different between low (2.0; 25-75%-ile: 1.75-2.0) and high-score (2.0; 1.0-2.75; *P* = 0.699). Intravascular ultrasound (IVUS) was used in 91% of low-risk patients and 100% of high-risk patients (*P* = 0.416). Spinal drains were used in 32% of low risk patients and 75% of high risk patients (*P* = 0.049). The number of patients requiring unplanned adjunct procedures were no different between low and high-risk groups (36% vs 50%; P = 0.678). Markers of procedural difficulty are listed in Table 6, showing patients in high-risk group had significantly greater blood loss (200 vs 100 mL; *P* = 0.038), fluoroscopy time (36.0 vs 16.6 min; *P* = 0.022), and a trend for increased procedure time (164 vs 95 min; *P* = 0.083) than patients in the low-risk group. Mean length of hospital stay was not significantly different between high and low-score groups (20.0 vs 15.5 days; *P* = 0.269). Kaplan-Meier survival analysis revealed no significant differences in all-cause mortality (log rank *P* = .80) or aortic-related mortality (log rank *P* = .56) between the high and low-score ASG groups (Figs. 5 & 6) However, Kaplan-Meier analysis revealed that the high-score group had a significantly decreased freedom from aortic-related reinterventions than the low-score group (38% vs 100% at 12-month followup; log rank *P* = 0.001; Fig. 7).

**Table 6:** Surgical variables (median; 25-75%-ile) relating to procedure difficulty compared between low and high score patient subgroups

	Low score (*N* = 22)	High score (*N* = 8)	*P*-value
Procedure time (min)	95 (78-159)	164 (101-235)	0.083
Blood loss (mL)	100 (100-163)	200 (175-525)	0.038
Contrast volume (mL)	172.4 (97.5-200)	149 (69.5-275)	1.000
Fluoroscopy time (min)	16.6 (11.6-22.5)	36.0 (32.6-44.6)	0.022

**Fig. 8:**
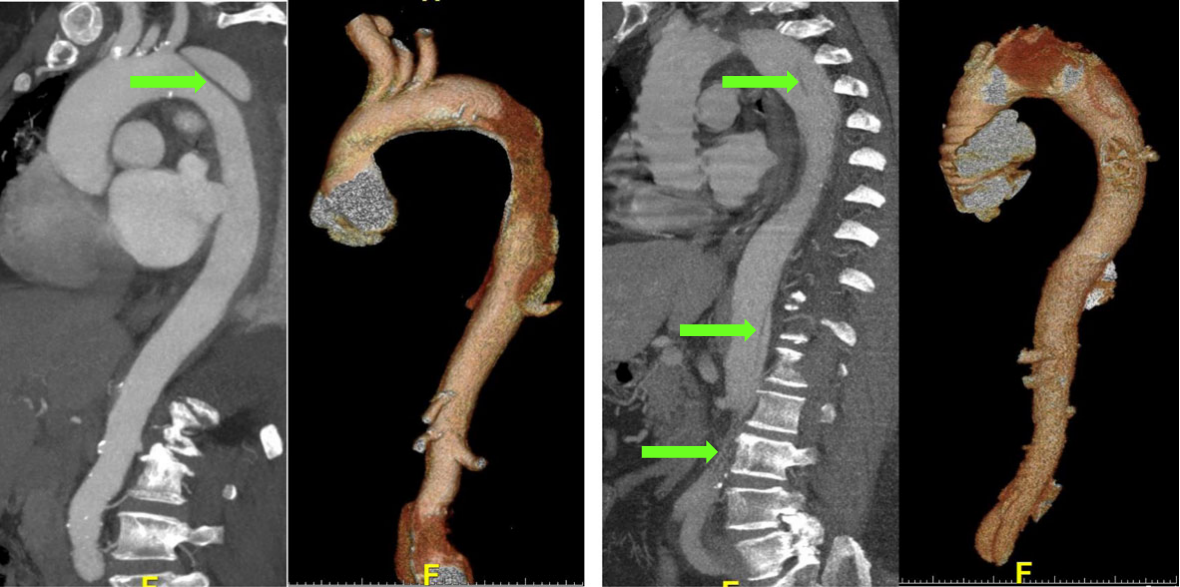
Representative CT imaging of a dissection patient in the low-score group (left; ASG = 10), and a patient in the high-score group (right; ASG = 29). Green arrows point to dissection flap

Representative computed tomography angiograms and 3D reconstructions of patients in the low and high-score groups are shown in Fig. 8. Mean ASG score of the low and high-score patient groups were 16.5 (SD: 3.8) and 24.4 (SD: 2.1), respectively. Of the 8 patients in the high-score group, 4 (50%) experienced aortic-related reinterventions, while of the 22 patients in the low-score group, only 1 (4.5%) experienced an aortic-related reintervention over the course of the study period (*P* = 0.011).

The mean thoracic aorta FLVI was 0.59 (range: 0.19-0.94). Patients in the high-score group exhibited significantly larger thoracic aortic FLVI (0.77 vs 0.53, *P* < 0.001). The correlation coefficient between thoracic aorta FLVI and ASG score was 0.74 (*P <* 0.001).

Multiple logistic regression was used to identify anatomic factors independently associated with aortic reinterventions. The two scoring parameters significant (*P* < 0.05) on univariate analysis were included in the multivariate model (Table 7). Regression analysis revealed patency of a branch vessel in the territory of the dissection communicated with the false lumen was independently associated with aortic reinterventions (OR: 0.120; 95% CI: 1.170-3.840; *P* = 0.013). The Hosmer-Lemeshow statistic for goodness-of-fit was not significant (*P* = 0.084), indicating that the logistic curve fit the equation.

**Table 7:** Multivariate analysis of anatomic morphology characteristics for aortic reinterventions

			Multivariate analysis		
	Attribute	Univariate analysis	*P*-value	Odds ratio	95% CI
Proximal Landing Zone	Whole lumen cross-sectional area contour change	0.404			
	Maximum whole lumen cross-sectional area	0.221			
	Left common carotid to left subclavian distance	0.312			
	Left common carotid to entry tear distance	0.394			
	Calcification and thrombus index	0.0345	0.122		
Curvature and Tortuosity	Thoracic aorta tortuosity index	0.0565			
	Intraaortic distance	0.821			
	Arch type	0.308			
Dissection	False lumen cross-sectional area index	0.133			
	False lumen length	0.540			
	Tear length	0.291			
	Number of observable tears	0.757			
	Tear circumferential location	1.000			
Branch Vessels	Location	0.576			
	Patency	0.00587	0.013	2.120	1.170-3.840
Supraceliac Aorta	Whole lumen cross-sectional area contour change	0.697			
	Maximum whole lumen cross-sectional area	0.445			

## Discussion

Various classification schemes have been proposed for aortic dissection including the University of Pennsylvania Classification of Acute Stanford Type-B Aortic Dissection (PENN-ABC) and the DISSECT mnemonic [6, 7]. However, neither of these classification schemes quantitatively risk-stratifies patients undergoing TEVAR for aortic dissections using a standardized numeric score. To our knowledge, this is the first scoring system that has been proposed to quantitatively evaluate anatomic factors relating to aortic-related reinterventions for patients undergoing TEVAR for acute TBAD. We sought to create a scoring system that could be used both as a clinical instrument to preoperatively evaluate the appropriateness of patients for TEVAR, as well as a research tool to standardize comparisons of patient populations across different studies.

The proximal landing zone component of the score sought to capture the difficulty associated with sealing the graft at the proximal landing zone, a critical requirement of successful TEVAR to prevent Type 1a endoleaks. We hypothesized that a larger aorta, larger variation in aortic cross-sectional area, and presence of calcification and thrombus would all potentially inhibit the successful apposition and seal of the proximal graft to the aortic wall. Numerous studies have demonstrated associations between false lumen and whole aorta size and aortic remodeling, morbidity, and mortality [8–10]. A smaller aortic diameter has been correlated with improved aortic remodeling after TEVAR for non-acute TBAD [11]. In addition, false lumen diameter at the upper descending thoracic aorta ≥ 22 mm was associated with a greater rate of aneurysmal change and mortality [10].

In addition, a shorter landing zone would both make it more difficult to accurately deploy the graft and also result in a smaller area of contact and thus form a more precarious and less durable seal between the graft and the inner wall of the aorta. In a study of patients treated surgically for type A dissections or medically for type B dissections, more proximal location of the entry tear, resulting in a smaller landing zone, was associated with increased dissection-related adverse events [8].

The purpose of the curvature and tortuosity component was to capture both difficulty of graft trackability through the aortic arch as well as graft seal associated with an aortic arch with a shorter radius and a thoracic aorta that is more tortuous. In addition, we hypothesized that a more angulated arch, as classified by the arch type, would lead to greater challenges with graft trackability and seal [12].

The dissection component of the score captured anatomic factors of the dissection itself, namely length and cross-sectional area of the false lumen, and the number and location of entry or reentry tears. We hypothesized that a longer dissection, a wider preoperative false lumen, and also larger or more numerous tears would negatively impact outcomes of TEVAR by impeding aortic remodeling because of false lumen patency and lead to an increased need for late aortic-related reintervention. In a study of patients treated surgically for type A dissections or medically for type B dissections, a larger entry tear was associated with greater risk for dissection-related adverse events [8]. We also hypothesized that due to what may be greater hemodynamic forces at the outer curvature of the arch as compared to the inner curvature, the location of the entry tear at the outer curvature would correlate with worsened outcomes. This is supported by studies demonstrating that in patients with non-acute TBAD, the absence of the primary entry tear at the outer curvature of the arch correlated with false lumen thrombosis following TEVAR [11]. In contrast, other studies have shown that location of the false lumen at the inner curvature is associated with increased aortic growth. However, these were performed on patients who were medically managed for TBAD rather than treated with TEVAR [4, 12].

We included a branch vessel component in the score because we believe that location of aortic branch vessels within the false lumen and occlusion of critical branch vessels might lead to ischemic complications that would worsen outcomes for these patients. Target organ or limb malperfusion is one of the major risk factors differentiating uncomplicated from complicated acute TBAD in PENN-ABC classification system for aortic dissections [13]. Branch vessel involvement can lead to end organ ischemia as well as increased propagation of the dissection proximally and distally, resulting in further proximal or distal malperfusion [14]. In addition, it is possible that presence of branch vessel ostia within the false lumen might inhibit thrombosis of the false lumen and resolution of the dissection, due to continued perfusion of the branch vessel or retrograde perfusion from the branch vessel. This is supported by studies showing that the absence of abdominal aortic branch vessel ostia in the false lumen was associated with complete obliteration of the false lumen after TEVAR [10, 15].

Finally, the supraceliac aorta score component was intended to capture the difficulty associated with larger aortic cross-sectional area and larger variation in aortic cross-sectional area in a segment of the aorta that most often serves as the distal landing zone of stent-grafts used in repairing dissections. The diameter of the false lumen and of the whole aorta at the level of the abdominal aorta has been shown to be a predictor of incomplete thrombosis of the false lumen after TEVAR [15].

We created the ASG score based both on a review of the literature and expert clinical opinion. Many of the components used in the ASG score are supported by previously published literature; however, it is difficult to assess the relative importance of each component due to the disparate methodologies of different studies. Therefore the weighing of various components of the score was based on reasoned judgment rather than a rigorous statistical analysis. As such, the score may be further refined by a regression analysis to determine the appropriate weight of each individual scoring component. Independent validation in a larger, prospective multi-center study with more diverse patient selection is needed to more accurately risk-stratify patients undergoing endovascular management of acute type B dissections.

A propensity analysis has been conducted for the effect of patient demographics, comorbidities and hospital characteristics on outcomes of TEVAR for TBAD, but this analysis did not examine anatomic factors [16].

Our study was limited by the small number of patients, especially in the high-score ASG group. The small cohort size limited our ability to stratify patients and potentially gain additional insight into how factors such as sex and race affect the validity of ASG score. Nevertheless, even with our modestly-sized study, we found a significant difference between the number of aortic-related reinterventions in the high score group versus the low score group, suggesting that this novel ASG score may be a strong predictor of aortic-related reinterventions.

A number of authors have advocated for volumetric measurements as a more accurate measure of pathologic aortic anatomy than diameter or length measurements [17, 18]. We correlated ASG scores with FLVI because we hypothesized that outcomes of TEVAR for dissection would be affected by the size of the false lumen, relative to the aorta as a whole. Volumetric analysis of true lumen and false lumen in aortic dissection before and after intervention has been used as a marker of disease course in patients treated with TEVAR and patients treated with medical management [19]. Volume has also been shown to be a predictor of disease progression and need for intervention. Prior studies from our group demonstrated that in patients presenting with acute uncomplicated TBAD, the ratio of true lumen volume to false lumen volume is inversely correlated with eventual intervention after the acute 14-day period [20]. Initial ratio of true lumen volume to false lumen volume was also inversely correlated with growth in aortic diameter between the time at presentation and follow-up imaging. Furthermore, in a study from our group on TEVAR for intramural hematoma, patients exhibited significant decreases in the volume of their intramural hematomas after intervention [21]. Therefore, volumetric analysis, while time consuming, appears to have utility as a correlate of various markers of disease severity in aortic dissection and the related pathology of intramural hematoma.

Our ASG score incorporated measures of the length and of the cross-sectional area of the false lumen – factors that can be expected to correlate with volume. However, directly measuring the volume of the false lumen is a time-intensive process, especially in patients with long and tortuous false lumens. Therefore, out of consideration for the practical use of the ASG score in clinic, we did not include direct volume measurements as a component of the score.

This ASG score represents a preliminary proposal and should be further validated with larger and more diverse patient groups. In addition, its sensitivity and specificity for reinterventions may be improved through further weighing of individual score components and testing against different patient populations at multiple centers.

## Conclusions

A preoperative ASG score of acute TBAD consists of analysis of the proximal landing zone, curvature and tortuosity of the aorta, dissection anatomy, aortic branch vessel anatomy, and supraceliac aorta anatomy. This numeric value can quantitatively describe the anatomopathologic complexity of acute TBAD. An ASG score ≥23 correlated with a larger false lumen to total aortic volume ratio and was an excellent predictor of aortic-related reinterventions. This ASG score should be further validated with larger and more diverse patient populations to increase its usefulness as a tool for clinical and research applications.

## Abbreviations

AAA: Abdominal aortic aneurysm
ASG: Anatomic severity grading
AUROC: Area under the receiver operating characteristic
EVAR: Endovascular aneurysm/aortic repair
FEVAR: Fenestrated endovascular aneurysm/aortic repair
FLVI: False lumen volume index
LCCA: Left common carotid artery
LSA: Left subclavian artery
PENN-ABC: Pennsylvania classification of acute stanford Type-B aortic dissection
SMA: Superior mesenteric artery
SVS: Society for vascular surgery
TBAD: Type B aortic dissection
TEVAR: Thoracic endovascular aortic repair
