# Erratum to: A novel anatomic severity grading score for acute Type B aortic dissections and correlation to aortic reinterventions after thoracic endovascular aortic repair

**DOI:** 10.1186/s13019-017-0616-2

**Published:** 2017-06-23

**Authors:** Shirui Chen, Sebastian Larion, Sadaf S. Ahanchi, Chad P. Ammar, Colin T. Brandt, Jean M. Panneton

**Affiliations:** Division of Vascular Surgery, Department of Surgery, Eastern Virginia Medical School, Sentara Heart Hospital, 600 Gresham Drive, Suite 8620, Norfolk, VA 23507 USA

## Erratum

The original article [1] contained an error in the Abstract whereby the symbol ‘≤’ was incorrectly used in the following sentence:

“Anatomic severity grading scores ≤23 are an excellent predictor of aortic-related reinterventions.”

The original article has now been updated so that the correct symbol, ‘≥’ is used instead to display the following corrected sentence:

“Anatomic severity grading scores ≥23 are an excellent predictor of aortic-related reinterventions.”
